# New Insights into the Antioxidant and Anti-Inflammatory Effects of Italian *Salvia officinalis* Leaf and Flower Extracts in Lipopolysaccharide and Tumor-Mediated Inflammation Models

**DOI:** 10.3390/antiox10020311

**Published:** 2021-02-19

**Authors:** Matteo Brindisi, Chouaha Bouzidi, Luca Frattaruolo, Monica R. Loizzo, Maria Stella Cappello, Annabelle Dugay, Brigitte Deguin, Graziantonio Lauria, Anna Rita Cappello, Rosa Tundis

**Affiliations:** 1 Department of Pharmacy, Health and Nutritional Sciences, University of Calabria, Via Pietro Bucci, 87036 Arcavacata di Rende, Italy; matteo.brindisi@unical.it (M.B.); luca.frattaruolo@unical.it (L.F.); monica_rosa.loizzo@unical.it (M.R.L.); graziantonio.lauria@unical.it (G.L.); rosa.tundis@unical.it (R.T.); 2 UFR de Pharmacie de Paris, Université de Paris, U.M.R. n°8038, CiTCoM-(CNRS, Université de Paris), F-75006 Paris, France; chouaha.bouzidi@parisdescartes.fr (C.B.); annabelle.dugay@parisdescartes.fr (A.D.); brigitte.deguin@parisdescartes.fr (B.D.); 3 CNR, Institute of Science of Food Production (ISPA), Prov. le Lecce-Monteroni, 73100 Lecce, Italy

**Keywords:** sage, HPLC-DAD-ESI-Q-MS, anti-inflammatory activity, antioxidant effects, cancer cell-conditioned media

## Abstract

This work aimed to investigate and compare the in vitro antioxidant and anti-inflammatory effects of *Salvia officinalis* L. (sage) from Italy, with the aim of raising its current knowledge in this field. Leaves and flowers (S1–S8), harvested in two areas of Southern Italy, were extracted with methanol as a solvent by maceration or ultrasound-assisted extraction. Sage extracts, analysed by high pressure liquid chromatography-diode-array detection-electrospray ionization-quadrupole-mass spectroscopy (HPLC-DAD-ESI-Q-MS), exerted a promising antioxidant activity investigated using ferric reducing antioxidant power (FRAP), 2,2-diphenyl-1-picrylhydrazyl (DPPH), 2,2′-azino-bis(3-ethylbenzothiazoline-6-sulphonic acid) (ABTS), and β-carotene bleaching tests, and elicited a significant decrease in reactive oxygen species (ROS) production in lipopolysaccharide (LPS)-stimulated RAW 264.7 murine macrophages. The anti-inflammatory activity was analysed in the same in vitro model. All the extracts did not affect cell viability although they showed anti-inflammatory activity, as they induced a decrease in nitrite levels that was greater than 50%, when employed at 50 µg/mL. Furthermore, they elicited a decrease in nitrite levels, as well as a decline in pro-inflammatory cytokine expression. The NF-κB transcription factor proved to be involved in the mechanisms that underlie such effects. Interestingly, sage extracts were able to interfere with the inflammatory activity induced by breast cancer cell-conditioned media (nitrite levels were significantly decreased, *p* < 0.05; *p* < 0.01), highlighting for the first time the important role of *S. officinalis* in controlling inflammation processes related to neoplastic progression.

## 1. Introduction

*Salvia* L. is one of the major genera in the Lamiaceae family. It includes about 900 species, many of which are used as spices or as flavouring agents in cosmetic industries [1]. Terpenoids and phenolic compounds are the most common and well-investigated classes of compounds that characterize the *Salvia* genus [2,3]. Other identified classes of compounds include tannins, alkaloids, fatty acids, and steroids [3]. Several *Salvia* species have shown interesting biological properties, including antioxidant [4,5], antimicrobial [6], anticancer [4], and anti-inflammatory effects [5,7].

*Salvia officinalis* L. (commonly known as sage) is a perennial, evergreen subshrub widely used in traditional medicine, native to the Mediterranean area, despite the fact that it has naturalized in many sites throughout the world. *S. officinalis* is one of the most widely appreciated plants due to its plethora of bioactive constituents that are mainly made up of terpenoids and phenolic acids [8]. A literature survey revealed that much of the phytochemical investigation in this species has focused on its essential oil. Such studies have identified various molecules, with the major constituents being camphor, 1,8-cineole, α-thujone, borneol, β-thujone, and viridiflorol [9]. However, other polar constituents have also been isolated, including carnosol, carnosic acid, rosmarinic acid, several flavonoids, and phenolic glycosides [8]. Many studies of this *Salvia* species have reported anti-cancer and anti-mutagenic properties related to several cancers including colon and breast cancer, as well as its antimicrobial, antidementia, hypoglycaemic, antinociceptive, antioxidant, and anti-inflammatory properties [2]. Kolac et al. [5] investigated the antioxidant and anti-inflammatory activities of *S. officinalis* in a lipopolysaccharide (LPS)-induced inflammation model. Superoxide dismutase, catalase, and glutathione peroxidase activities in the inflammation group were significantly higher in the group treated with *S. officinalis*. The inflammation group showed increased levels of nitric oxide (NO), tumor necrosis factor-α (TNF-α), and nuclear factor NF-κB. *S. officinalis* showed promising effects on LPS-induced inflammation and oxidative stress in rats. Terpenes and polyphenols (including flavonoids) are the constituents of sage that most probably contribute to the antinociceptive and anti-inflammatory effects of this *Salvia* species [10]. Flavonoids isolated from *S. officinalis* reduced inflammation in the mouse carrageenan model and showed analgesic effects [10]. Previously, Osakabe et al. [11] showed that rosmarinic acid, one of the most abundant compounds that characterize the phytocomplex of *S. officinalis*), topically used inhibited epidermal inflammation.

Over the years, increasingly advanced extraction techniques have been developed and optimized which have improved the quality and specificity of extracts. However, when dealing with plants, a key role, regarding the yield and composition in phytocomplexes, is played by the conditions and/or the place and season of collection, as well as the part of plant used for extraction [8]. In this context, herein, the chemical profiles of *S. officinalis* extracts obtained by ultrasound-assisted extraction and maceration of leaves and flowers, collected in two areas of Southern Italy (Orsomarso and Civita) were analysed. Subsequently, their antioxidant and anti-inflammatory effects were assessed. We focused this research on oxidative stress and inflammation, two topics which have aroused increasing attention in recent years. Oxidative stress is reported as an imbalance between both free radicals and reactive oxygen species (ROS) production, and their elimination is mediated by antioxidant mechanisms. This imbalance elicits severe biomolecular and cellular damage, inducing potential effects on the whole organism. In the last decade, various studies have demonstrated that continuous oxidative stress is able to trigger the inflammatory process which, if it becomes chronic, can mediate many diseases, including cancer. Several reports support the link between the oxidative state, inflammatory process, and cancer, which is further confirmed by the anti-tumor efficacy displayed by anti-inflammatory and antioxidant therapy [12,13,14]. The close relationship between inflammation and neoplastic onset/progression, prompted us to assess whether *S. officinalis* extracts from Orsomarso and Civita were able to interfere in the inflammatory microenvironment produced by tumor cells. To this end, for the first time, we investigated the potential effects of *S. officinalis* extracts in reducing the inflammation related to breast cancer cell progression.

## 2. Materials and Methods

### 2.1. Chemicals and Reagents

All the solvents of analytical grade were purchased from VWR International s.r.l. (Milan, Italy). The liquid chromatography-mass spectrometry (LC-MS) quality grade methanol and acetonitrile were obtained from Carlo Erba (Val de Reuil, France). Ultrapure water (18.2 MΩ.cm) was obtained from Elga and Purelab Classic (Veolia Water, Antony, France). Formic acid (used at 10 g/L) was provided from Carlo Erba (Val de Reuil, France). Standard molecules, namely rutin (purity >99%), carnosol (purity >90%), luteolin-7-glucoside (purity >98%), ursolic acid (purity >98%), rosmarinic acid (purity >99%), and carnosic acid (purity >90%) were purchased from Extrasynthese (Genay, France). The Folin-Ciocalteu reagent, ascorbic acid, 2,2-diphenyl-1-picrylhydrazyl (DPPH), quercetin, propyl gallate, linoleic acid, chlorogenic acid, β-carotene, dimethyl sulfoxide (DMSO), sodium carbonate, butylated hydroxytoluene (BHT), 2,2′-azino-bis (3-ethylbenzothiazoline-6-sulfonic acid) diammonium salts (ABTS), Fetal Bovine Serum (FBS), and Tween 20 were purchased from Sigma-Aldrich S.p.a. (Milan, Italy).

### 2.2. Plant Materials

*S. officinalis* leaves and flowers were collected in June 2018 in two localities of Southern Italy, in particular in Orsomarso (39°48′ N, 15°55′ E, 240 m above sea level) (voucher specimen n. CLU 26259), and in Civita (39°50′ N, 16°19′ E, 620 m above sea level) (voucher specimen n. CLU 26262). Samples have been identified by Dr. NG Passalacqua, Natural History Museum of Calabria and the Botanic Garden (University of Calabria, Italy). In each locality, aerial parts were harvested in order to obtain adequate amounts for the chemical and biological analysis. Both leaves and flowers were examined to verify the absence of insect contamination and dust as well as the integrity.

### 2.3. Extraction Procedure

Fresh leaves and flowers of *S. officinalis* were subjected to two extraction procedures using methanol as a solvent: 1) Maceration (1 L, 3 × 72 h), and 2) ultrasound-assisted extraction by using the Branson 3800 ultrasonic system, series CPXH (130 W, 40 kHz frequency (Milan, Italy) (150 mL, 3 × 1 h). In Table 1, the extraction yields are reported.

### 2.4. Total Phenol Content (TPC)

The Total Phenol Content (TPC) was determined using the Folin-Ciocalteu reagent [15]. Absorbance was read at 765 nm using a UV-Vis Jenway 6003 spectrophotometer (Milan, Italy) and TPC was expressed as mg of chlorogenic acid equivalents (CA)/g of plant materials.

### 2.5. Total Flavonoid Content (TFC)

To assess the total flavonoid content (TFC), a previously described spectrophotometric method was employed [15]. Absorbance was measured at 510 nm and TFC was expressed as mg of quercetin equivalents (QE)/gram of plant materials.

### 2.6. High Pressure Liquid Chromatography-Diode-Array Detection-Electrospray Ionization-Quadrupole-Mass Spectroscopy (HPLC-DAD-ESI-Q-MS) Compounds Profiling

The extracts of *S. officinalis* (S1–S8) were analysed using an HPLC-DAD-MS ThermoScientific Dionex U3000 (Thermo-Dionex, Les Ulis, France) including a quaternary pump (LPG-3400 SD), a thermostat column (TCC-3000SD), a thermostat autosampler (WPS-3000TSL), and a diode array detector (DAD-3000) in line with a quadrupole mass spectrometer (Surveyor MSQ plus System, Thermo-Dionex, Les Ulis, France). The analytical column was a C18 Acclaim Polar advantage II (3 µm, 120 Å, 100 × 2.1 mm i.d.) (Dionex Bonded Silica Products, Les Ulis, France) heated to 35 °C during the analyses. The pump flow rate was 0.5 mL/min. The injection volume was 20 µL.

Two chromatographic mobile phases were used. Eluent A was formic acid (1%, *V/V*) in water and eluent B was formic acid (1%, *V/V*) in acetonitrile. The binary gradient was formulated as follows: 5% eluent B during 5 min then, in 10 min eluent B reached 20%, and in 5 min eluent B reached 25%, in 10 min eluent B reached 30%, in 10 min eluent B reached 40%, in 5 min eluent B reached 50%, in 5 min eluent B reached 80%, then stayed at 80% during 3 min before coming back to the initial conditions in 2 min.

**Figure 1:**
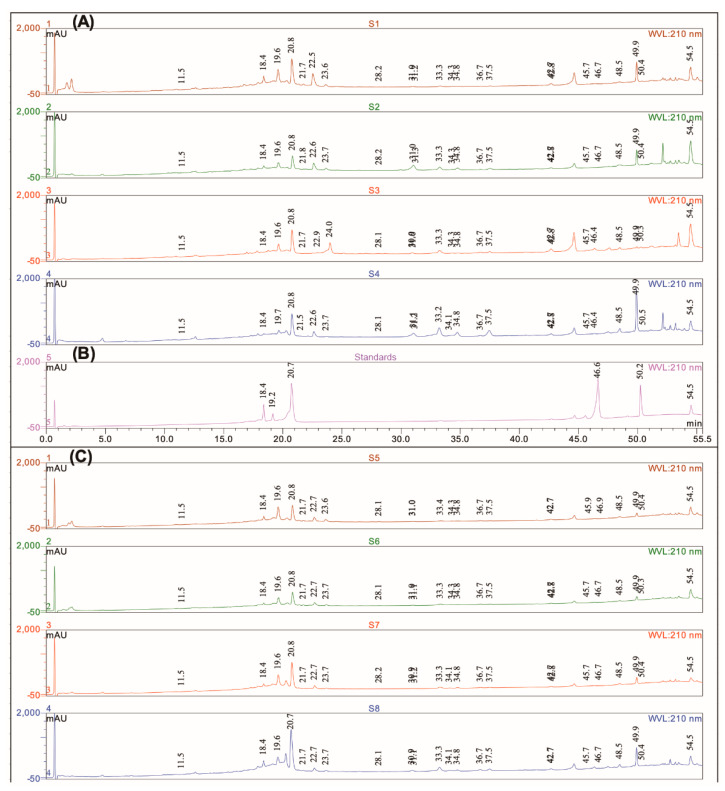
Compounds identification at 210 nm: Profiles of extracts of *S. officinalis* from Orsomarso: (**A**) S1, S2, S3, and S4. (**B**) The mixture of standards is obtained by diluting to one fifth of the solution initially prepared: Luteolin-7-glucoside (0.052 g/L, retention time (Rt) = 18.4 min), rosmarinic acid (0.194 g/L, Rt = 20.7 min), rutin (0.032 g/L, Rt = 19.2 min), carnosol (0.102 g/L, Rt = 46.6 min), carnosic acid (0.082 g/L, Rt = 50.2 min), and ursolic acid (0.142 g/L, Rt = 54.5 min). Profiles of *S. officinalis* extracts from Civita: (**C**) S5, S6, S7, and S8.

UV spectra were performed using a diode array detector with a wavelength scanning between 200 and 400 nm. Detection at definite wavelengths such as 210, 254, 280, and 350 nm, was used to record the chromatograms. The chromatographic effluent carried by a stream of nitrogen was directed into the electrospray ionization source (ESI) of the mass spectrometer (MS). The MS was operated in the positive and negative ionization modes with the following conditions: Ion spray voltage 3 kV, curtain gas 50 psi, Q energy was 70 V, cone voltage 50 V, desolvation temperature 500 °C, and ion energy 0.8 V. In all cases, mass spectra were acquired in the range of 100–1000 Th. Chromeleon®, version 6.8 software provided by Thermo Scientific Dionex (Les Ulis, France) was used for the treatment of the results. All the experiments were conducted successively over 3 days, in triplicates each time. Finally, compounds present in the sage extracts have been identified and quantified using a methanolic standard solution of pure compounds previously described in *S. officialis* such as rutin, rosmarinic acid, ursolic acid, and luteolin-7-glucoside [16,17]. Standard chromatograms recorded after the HPLC-DAD-ESI-Q-MS experiments are reported in Figure 1. For these experiments, the following solutions have been prepared: Carnosol (0.51 g/L), luteolin-7-glucoside (0.26 g/L), carnosic acid (0.50 g/L), rosmarinic acid (0.97 g/L), ursolic acid (0.71 g/L), and rutin (0.16 g/L). Calibration curves used a linear fitting (unweight and not forced to axis-origin) in the concentrations range of 0.008–0.4 g/L. A coefficient determination R2 > 0.99 was used as an acceptability threshold for calibration purposes. The results were expressed as g/L. Carnosol and carnosic acid have not been evaluated as they are not stable over time as previously indicated [12]. *S. officinalis* extracts were dissolved in methanol before being analysed in HPLC-DAD-MS:S1 (1.86 g/L); S2 (1.47 g/L); S3 (2.24 g/L); S4 (2.04 g/L); S5 (1.92 g/L); S6 (1.46 g/L); S7 (2.07 g/L), and S8 (2.24 g/L).

### 2.7. In Vitro Antioxidant Activity

*S. officinalis* extracts (S1–S8) have been investigated for their potential antioxidant effects by applying four assays namely Ferric Reducing Antioxidant Power (FRAP), ABTS, β-carotene bleaching, and DPPH tests following the procedures previously described [15].

In the ABTS test, after 12 h of incubation, the ABTS solution was diluted with methanol to a final absorbance of 0.70 at 734 nm. Sequentially, 2 mL of the diluted ABTS solution was added to the sage extract at concentrations in the range 1–400 µg/mL. After 6 min, the absorbance was read at 734 nm. In the DPPH test, *S. officinalis* extracts at different concentrations (62.5–1000 µg/mL) were added to the DPPH solution. After 30 min of incubation, the absorbance was measured at 517 nm. Ascorbic acid was selected as a positive control in both ABTS and DPPH tests. The ability to reduce iron ions of sage extracts was examined using the FRAP test. Samples were tested at the concentration of 1 mg/mL and the FRAP value was expressed as µM Fe(II)/g. The absorbance was read at 595 nm. BHT (butylated hydroxytoluene) was used as a positive control. The potential of sage extracts to inhibit lipid peroxidation was evaluated using the β-carotene bleaching test. Samples were tested at different concentrations that ranged from 100 to 2.5 µg/mL and propyl gallate was used as a positive control.

### 2.8. Cell Cultures

RAW 264.7 cell line and breast cancer cells (MCF7 and MDA-MB-231) were acquired from the American Culture Collection (ATCC, Manassas, VA, USA). RAW 264.7 cells were grown in DMEM (Sigma-Aldrich, St. Louis, MO, USA) supplemented with 2 mM L-glutamine (Gibco, Life Technologies, Waltham, MA, USA), 10% Fetal Bovine Serum (FBS, Sigma-Aldrich, St. Louis, MO, USA), and 1% penicillin/streptomycin (Gibco, Life Technologies, Waltham, MA, USA), whereas MCF7 and MDA-MB-231 were cultured in DMEM/F12 (Sigma-Aldrich, St. Louis, MO, USA) supplemented with 1% penicillin/streptomycin (Gibco, Life Technologies, Waltham, MA, USA) and 10% FBS, as reported previously [18]. All the cell lines were maintained at 37 °C in a humidified atmosphere (5% CO_2_).

### 2.9. Reactive Oxygen Species (ROS) Assessment

ROS were quantified using the chloromethyl derivative of 2’,7’-dichlorodihydrofluorescein diacetate (CM-H_2_DCFDA, Thermo Fisher Scientific, Waltham, MA, USA), as previously described [19,20]. Briefly, 2x10^5^ RAW 264.7 cells/well were seeded in 6-well plates and simultaneously treated for 1 h with extracts, at their IC_50_ values_,_ and LPS (1 µg/mL). Then, the treated cells were rinsed with PBS, harvested, resuspended in 5 μM CM-H_2_DCFDA, a fluorescent probe used as an indicator for ROS, in PBS and incubated at 37 °C, for 45 min. Subsequently, the stained cells were harvested by centrifugation and maintained in a fresh medium. Lastly, each fluorescence sample was quantified by fluorimetry (Synergy H1 microplate reader, BioTek, Winooski, VT, USA), and its intensity was normalized by a viable cell number (automated cell counter, TC20, Bio-Rad, Hercules, CA, USA).

### 2.10. NO Production in LPS-Stimulated RAW 264.7 Cells

Stable oxidized products of NO in cell culture media were assessed by the Griess reagent, as already reported [21,22]. The 2x10^5^ RAW 264.7 cells/well were seeded in 24-well plates and cultured overnight, in the complete medium. Then, the cells were treated for 24 h with 1 µg/mL LPS and extracts, at increasing concentrations. DMSO (Sigma-Aldrich, St. Louis, MO, USA) was used as a control. Next, a cell culture supernatant share (100 µL) was mixed with an equal volume of the Griess reagent in a 96-well plate and subjected to spectrophotometric analysis at 550 nm using a microplate reader (Synergy H1 microplate reader, BioTek, Winooski, VT, USA).

### 2.11. Cell Viability Assay

Cell viability was assessed using the 3-(4,5-Dimethyl-2-thiazolyl)-2,5-diphenyl-2H-tetrazolium bromide (MTT, Sigma-Aldrich, St. Louis, MO, USA) assay, as previously described [23,24]. In brief, RAW 264.7 cells were exposed to increasing concentrations of each extract for 24 h. Next, the MTT solution was put into each well (to a concentration of 0.5 mg/mL) and plates were maintained for 2 h at 37 °C. Then, the formazan crystals formed in each well were solubilized in DMSO and spectrophotometrically quantified at 570 nm, using a microplate reader.

### 2.12. Immuno-Fluorescence Monitoring Nuclear Factor Kappa B (NF-κB) Translocation

Murine macrophages were spread on coverslips in 6-well plates at 1 × 10^5^ cells/well of density, and grown in a complete medium, overnight. Then, the cells were exposed for 1 h to 1 µg/mL LPS and extracts, at their IC_50_. Subsequently, cells were fixed for 20 min with methanol at −20 °C, rinsed three times with a Tris-buffered saline (TBS, Sigma-Aldrich, St. Louis, MO, USA) for 5 min, and blocked with 5% bovine serum albumin (BSA, Sigma-Aldrich, St. Louis, MO, USA) in TBS at 37 °C, for 40 min. After incubation at 37 °C, for 40 min, with the anti-NF-κB p65 monoclonal antibody (Santa Cruz, Biotechnology, Dallas, TX, USA), as previously indicated [19,25], cells were rinsed three times for 5 min with TBS, to discard the excess of primary antibody and incubated at 37 °C, for 40 min, in anti-mouse IgG-TRITC (Thermo Fisher Scientific, Waltham, MA, USA). Finally, they were washed with TBS three times for 5 min. The 20× magnification images were acquired on an Olympus BX41 microscope with the CSV1.14 software. The image acquisition was performed using a CAMXC-30.

### 2.13. Quantitative PCR Analysis

RAW 264.7 cells were grown in 10 cm dishes to 70–80% confluence and for 6 h were treated with DMSO, with 1 µg/mL LPS alone or in the presence of extracts at their IC_50_ values. The TRIZOL reagent (Sigma-Aldrich, St. Louis, MO, USA) was used to extract total cellular RNA, according to the manufacturer’s instructions. RNA purity and integrity were verified by both spectroscopical analysis and gel electrophoresis. The synthesis of complementary DNA (cDNA) was obtained, via reverse transcription. The gene expression analyses of interleukin 1 beta (IL-1β), tumor necrosis factor alpha (TNFα), and interleukin 6 (IL-6) were achieved by the Quant Studio7 Flex Real-Time PCR System platform (Life Technologies, Waltham, MA, USA) using the SYBR Green Universal PCR Master Mix (Roche, Monza, MB, Italy), as previously described [12]. Experiments were performed in triplicates and expression levels of the studied genes were normalized onto the Glyceraldehyde 3-phosphate dehydrogenase (GAPDH) mRNA levels. Relative mRNA levels were calculated using the ∆∆Ct method and comparing it with the control group. In Table 1, the primers utilized are grouped here.

### 2.14. Statistical Analysis

Experiments were performed in triplicates and data are expressed as the means ± standard deviation (SD). The concentration giving 50% inhibition (IC_50_) was calculated by nonlinear regression using the GraphPad Prism version 8.0 (GraphPad Software, San Diego, CA, USA). ANOVA followed by Dunnett’s test was applied to evaluate the differences between data and positive control results in biological assays. Tukey’s test was used to determine any significant difference between all the treatments at different levels. The Relative Antioxidant Capacity Index (RACI) was used to establish the antioxidant potential [26].

## 3. Results and Discussion

### 3.1. Chemical Profile

Considering the healthy properties of sage, the choice of efficient extraction procedures is drawing great attention and solvent extraction is the most used procedure.

Herein, we have extracted the fresh leaves and flowers of *S. officinalis* collected in two localities of Calabria (Southern Italy) by maceration and ultrasound-assisted extraction by using methanol as a solvent in both extraction procedures. Table 2 reports the extraction yield (%). The analysis of the results evidenced that maceration was more effective (9.3–7.8%) than ultrasound-assisted extraction (8.4–3.8%) using the same solvent.

The phytochemical composition of *S. officinalis* extracts was determined using the HPLC-DAD-ESI-Q-MS mass spectrometric method. The identification of carnosic acid, rosmarinic acid, carnosol, luteolin-7-glucoside, and ursolic acid were confirmed using authentic standards (Figure 1B). Calibration curves used a linear fitting in the concentrations range of 0.008–0.4 g/L. A coefficient determination R^2^ > 0.99 was used as an acceptability threshold for calibration purposes. The other *S. officinalis* constituents were identified based on retention time (Rt), wavelengths of maximum absorption in the UV-VIS region, and molecular weight (*m*/*z* ion [M + H]^+^ and [M − H]^−^), in comparison to the spectral data concerning the phytochemical profile of *S. officinalis* previously published [16,17,27,28,29]. Chromatograms allowed the putative annotation of 22 compounds as reported in Figure 1A,C, and in Table 3.

A very good similarity between the chromatograms is to be noted. Rosmarinic acid (Rt = 20.8 min), carnosic acid (Rt = 50.4 min), ursolic acid (or isomers [12]), Rt = 54.5 min), and luteolin-7-glucoside are identified in all the flower and leaf extracts. However, some differences or characteristics can be shown.

Luteolin-7-glucoside is present in all the extracts with a small area and not detected in the S3 extract. In the leaves or flowers, triterpenic acids are the most abundant compounds dosed, then rosmarinic acid, and finally luteolin-7-glucoside is the minority dosed compound. Luteolin-7-glucoside, rosmarinic acid, and triterpenic acids quantification is reported in Table 4.

The concentration of the selected compounds varied according to the extraction method with significant differences. Except for *S. officinalis* from Civita, methanolic maceration is more favourable for the extraction of luteolin-7-glucoside and rosmarinic acid from the leaves. For the extraction of triterpenic acids such as ursolic acid [12], a more efficient extraction was obtained using the ultrasound technique.

The ultrasound-assisted extraction was more successful than the classical maceration in extracting luteolin-7-glucoside and rosmarinic acid of the flowers. The same trend is observed for triterpene acids of the flowers from Civita.

### 3.2. Antioxidant Effects

Oxidative metabolism is indispensable for the cells survival. A side effect of this is the production of reactive oxygen species (ROS), which can induce oxidative changes.

When there is an excess of free radicals, ROS can overwhelm protective enzymes and cause destructive and lethal cellular effects by oxidizing DNA, lipids, and proteins. Oxidative stress originates from an imbalance in the antioxidant status.

Among the endogenous defences are enzymes such as superoxide dismutase, glutathione peroxidase, and catalase. In addition to these defences, the consumption of dietary antioxidants is very important. The antioxidant activity cannot be evaluated reasonably by one antioxidant assay without due regard to the several variables that influence the results. Different tests may be required to assess such antioxidant effects. For these reasons, herein sage extracts were analysed using four assays such as FRAP, ABTS, β-carotene bleaching, and DPPH tests. Results (IC_50_ values) are reported in Table 5.

In the DPPH assay, the most interesting results were observed with samples S3 and S7, namely the extracts obtained by maceration of the flowers from Orsomarso and Civita, respectively, with the IC_50_ value of 9.8 μg/mL. Both samples are characterized by the highest TPC (41.9 and 41.6 mg/g plant materials for S3 and S7, respectively). Interesting results were also obtained with S2 and S5 with IC_50_ values of 10.4 and 10.3 μg/mL, respectively.

In the ABTS test, except for S5, all the sage extracts (IC_50_ in the range 0.9–1.2 μg/mL) are more active than ascorbic acid (IC_50_ of 1.7 μg/mL), used as a positive control.

The extract with the greatest ability to protect the oxidation of β-carotene is S6 with IC_50_ of 2.0 and 2.5 μg/mL after 30 and 60 min of incubation, respectively. Comparable values were obtained with S3 with IC_50_ values of 2.9 and 2.5 μg/mL after 30 and 60 min of incubation, respectively.

In the FRAP test, sage extracts are more active than the positive control BHT. The most promising are the extracts S1, S3, and S8 with values of 98.4, 95.5, and 94.4 μM Fe(II)/g.

In Table 5, the total phenols content (TPC) and total flavonoids content (TFC) are also reported. Generally, extracts obtained by maceration exhibited the highest TPC and TFC than samples obtained by ultrasound-assisted extraction. The highest content of phenols was found in samples S3 and S7 with values of 41.9 and 41.6 mg CA equivalents/g plant materials, respectively. S7 and S1 were the richest extracts in flavonoids with values of 26.6 and 25.2 mg QE equivalents/g plant materials, respectively.

**Figure 2:**
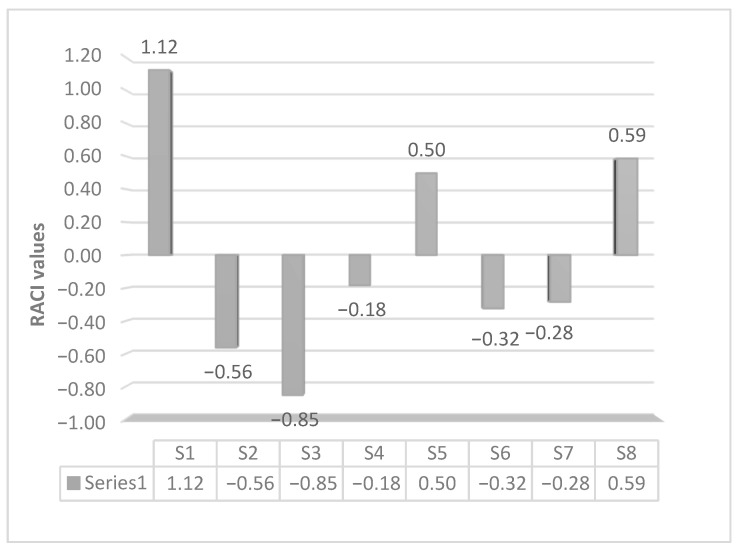
Relative antioxidant capacity index (RACI) values of sage extracts (S1–S8).

The statistical approach relative antioxidant capacity index (RACI) was used to establish the antioxidant rank of *Salvia* extracts (Figure 2). Sample S3 showed the highest activity followed by the S2 and S6 extracts.

Several works have shown *S. officinalis* to be one of the main sources of potent antioxidants [30]. The antioxidant effects of *S. officinalis* were mainly related to the presence of carnosic acid and rosmarinic acid. Rosmarinic acid showed strong antioxidant effects in several *in vitro* and *in vivo* studies [31].

Rosmarinic acid was demonstrated to suppress ROS generation and inhibit lipids peroxidation. Moreover, it showed free radicals scavenging effects in hepatic stellate cells as a result of its antioxidant activity by increasing glutathione synthesis and by participating in the NF-κB-dependent inhibition of matrix metalloproteinase-2 activity. This compound increased the catalase, glutathione peroxidase, and superoxide dismutase activities [31]. However, other works on *S. officinalis* have reported the presence of numerous diterpenoids, phenolic acids, and flavonoids that demonstrated a remarkable antioxidant activity including sage constituents identified in our samples such as carnosol, carnosic acid, luteolin, caffeic acid, 7-*O*-methylrosmanol, apigenin, rosmadial, pedalitin, 12-*O*-methylcarnosic acid, apigenin-*O*-glucuronide, luteolin-7-glucoside, luteolin-*O*-glucuronide, nepetin, hispidulin, cirsimaritin, and genkwanin [32].

Carnosic acid and carnosol were able to protect the lipids from oxidation. Loussouarn et al. [33] showed that upon the ROS oxidation of lipids, carnosic acid was consumed and oxidized into several compounds that include carnosol, whereas carnosol resisted. This suggested that carnosic acid is a chemical quencher of ROS. Carnosol occurs in the lipid oxidation process. Under oxidative conditions that did not include the ROS generation, contrary to carnosic acid, carnosol inhibited lipid peroxidation. [33].

### 3.3. S. officinalis Extracts Reduce ROS Levels

Oxidative stress exerts a crucial role in the initiation and progression of various pathologies including cancer. Of all the species produced under such conditions, ROS are the most abundant [34]. The ROS intracellular concentration, as well as the balance between ROS and endogenous antioxidant compounds can induce cellular impairment, damaging proteins, lipids, RNA, and DNA [35].

**Figure 3:**
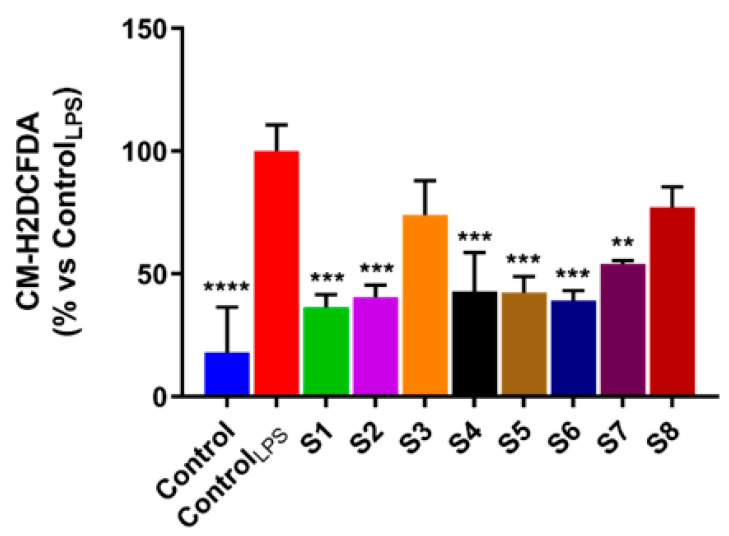
*Salvia officinalis* extracts decrease the reactive oxygen species (ROS) in lipopolysaccharide (LPS)-stimulated RAW 264.7 cells. Cells were exposed to *S. officinalis* extracts (S1-S8), for 24 h; ROS intracellular levels were assessed, using the CM-H2DCFDA dye, as reported in Materials and Methods. Values are the mean ± SD of three independent experiments, each one performed in triplicate. *p*-values were calculated vs. control_LPS_; ** *p* < 0.01; *** *p* < 0.001; **** *p* < 0.0001.

Natural antioxidants have been reported to display antioxidant activity, reducing intracellular ROS production, thus preventing damage caused by high oxidative stress conditions [36]. In this context, several research groups have proved that *S. officinalis* extracts endowed with a powerful antioxidant activity manage to prevent many of the effects mediated by oxidative stress, such as DNA damage [37,38]. This effect was promptly ascribed to the massive presence in phytocomplexes of compounds with a remarkable antioxidant activity. These literature data, as well as the promising antioxidant activity exhibited by leaves and flowers of *Salvia officinalis* extracts in our different antioxidant in vitro assays, prompted us to evaluate whether these extracts were able to manifest antioxidant activities in the cell. In detail, based on the close connection between oxidative stress and inflammation [39], we evaluated intracellular ROS levels, in LPS-stimulated RAW 264.7 murine macrophage-like cells widely used as an *in vitro* model to assess the inflammatory process. This system allowed us to highlight, for the first time, that *Salvia officinalis* extracts were able to reduce oxidative stress induced by an inflammatory stimulus. Indeed, murine macrophage-like cells stimulated with LPS displayed increased intracellular levels of radical oxygen species (Figure 3). Instead, after treatment with *Salvia officinalis* extract, they exhibited significant decreases in ROS production, with the exception of those treated with S3 and S8 (Figure 3). This confirms the good antioxidant activity exhibited by the extracts, even at the cellular level, in the above in vitro antioxidant assays, highlighting the ability of *S. officinalis* species to reduce the oxidative stress induced by an inflammatory process.

### 3.4. S. officinalis Extracts Reduce NO Production Inhibiting NF-κB

**Figure 4:**
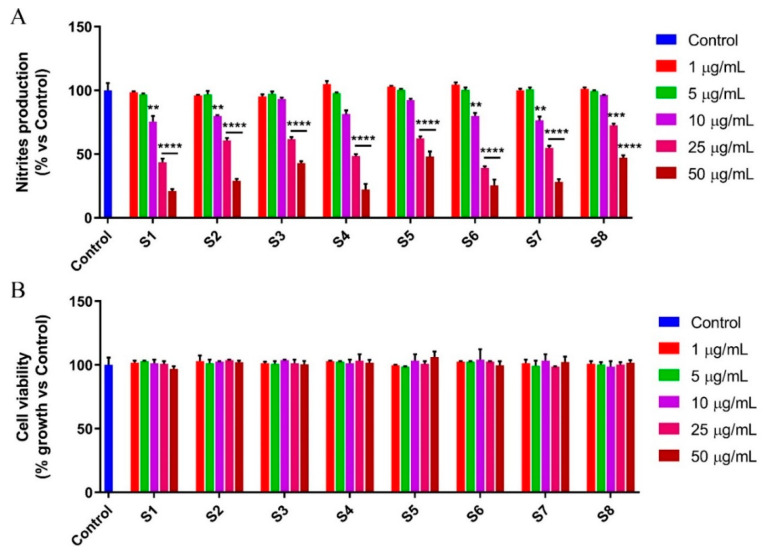
S1–S8 extracts significantly reduce nitric oxide (NO) production. Nitrites production (**A**) and cell viability (**B**) were assessed by the Griess and MTT assays, respectively, after 24 h of treatment of the LPS-stimulated RAW 264.7 cell line with sage extracts, at increasing concentrations (from 1 to 50 µg/mL). The results of the Griess assay are expressed as a percentage of nitrites production vs. control, while the MTT assay results are indicated as a percentage of cell viability vs. control. Values are the mean ± SD of three independent experiments, each one performed in triplicate. *P*-values were calculated vs. control; ** *p* < 0.01; *** *p* < 0.001; **** *p* < 0.0001.

It is known that different inflammatory stimuli, such as LPS, can trigger several inflammatory diseases by regulating nitric oxide (NO) levels [39]. Indeed, in LPS-stimulated cells, NO exerts a pivotal role in the inflammatory process. Based thereon, and given the many compounds endowed with potential anti-inflammatory activity detected in the S*alvia officinalis* leaf and flower extracts tested, we next evaluated their ability to reduce the NO release (Figure 4A,B).

First, we performed the MTT assay on LPS-stimulated RAW 264.7 cells, in order to assess the effect of increasing concentrations of the different extracts (from 1 to 50 µg/mL) on cell viability. The results demonstrated that the treatment does not induce any significant decrease in cell growth (Figure 4B). Then, the murine macrophage-like cells were stimulated with LPS and treated with the extracts of *Salvia officinalis* leaves and flowers, at the same concentrations previously used. After 24 h of treatment, the Griess assay, performed on the culture medium, showed a marked reduction in NO using all the extracts tested, in a dose dependent manner (Figure 4A and Table 6).

**Figure 5:**
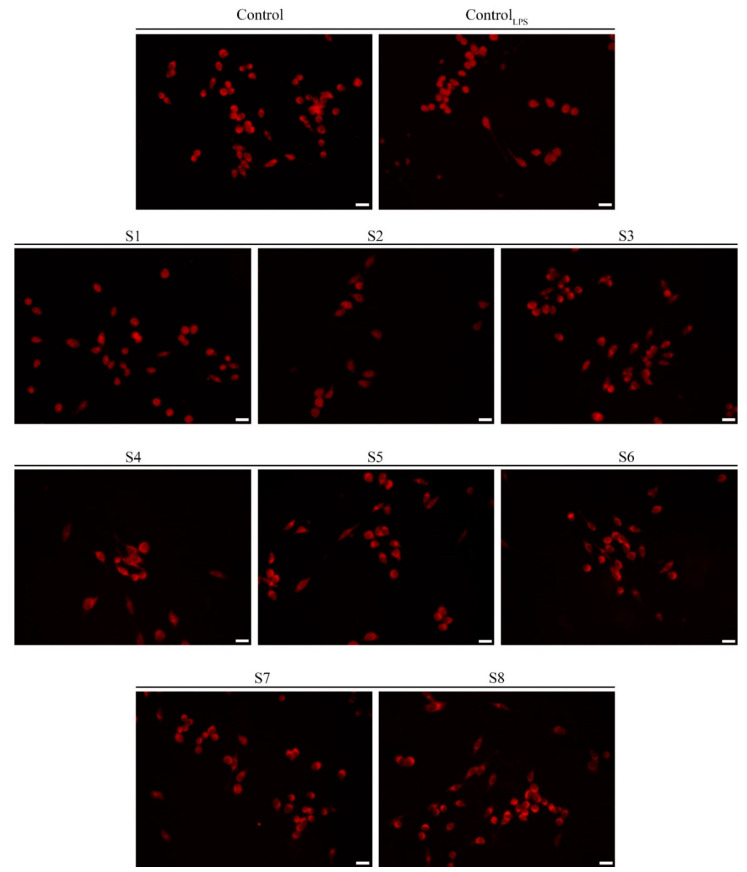
*Salvia officinalis* extracts interfere with the NF-κB nuclear translocation in RAW 264.7 cells. Immuno-fluorescent detection of NF-κB in cells treated with DMSO (control) or 1 μg/mL LPS + DMSO (control_LPS_) or 1 μg/mL LPS + S1–S8 extracts (as indicated), at their IC_50_, for 1 h. Scale bar: 50 μm.

These outcomes are in agreement with the literature data, which has evidenced a wide range of pharmacological properties for *S. officinalis*, including the anti-inflammatory activity, due to their chemical constituents [40]. Accordingly, an interesting activity in the modulation of inflammatory processes can be ascribed to the extracts of *Salvia officinalis* leaves and flowers from Orsomarso and Civita. Furthermore, these results, together with the noticeable antioxidant activity elicited by S1–S8 extracts, laid the foundation for assessing a probable involvement of the NF-κB transcription factor in the mechanisms that underlie these effects. NF-κB acts as a pivot in inflammatory and oxidative stress response by inducing the inducible nitric oxide synthase expression which, in turn, catalyses the NO production [19,25]. The translocation of NF-κB from the cytosol to the nucleus determines the transcription of genes correlated with an increase in the oxidative and inflammatory state [19]. Thus, the nuclear translocation in LPS-stimulated murine macrophages was evaluated, after the S1–S8 exposure (Figure 5).

The results evidenced that murine macrophage-like cells not stimulated with LPS were characterized by fluorescence delimited in the cytosol, confirming the inactivation in the basal state of NF-κB signaling. By contrast, the fluorescence moved to the nucleus when the murine macrophage-like cells were exposed to LPS, underlining the translocation of NF-κB into the nucleus. As shown in Figure 5, the exposure of LPS-stimulated RAW 264.7 cells to *Salvia officinalis* extracts (S1–S8) evidenced a very similar condition to the basal one, in which the fluorescence was confined, to a great extent, into the cytosol.

### 3.5. S. officinalis Extracts Reduce Pro-Inflammatory Cytokines

**Figure 6:**
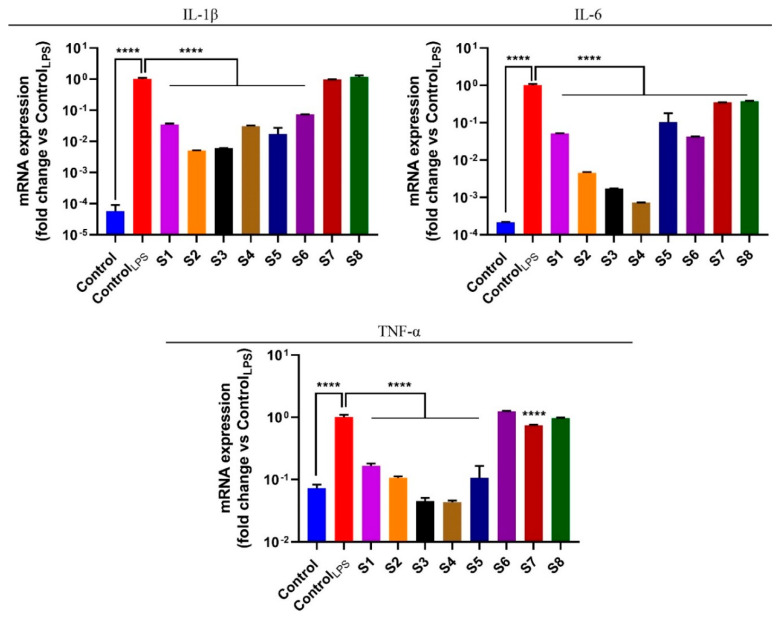
S1–S8 extracts and modulates regulate pro-inflammatory cytokines expression levels in LPS-stimulated RAW 264.7 cells. Effects are induced by the S. officinalis extracts treatment on mRNA levels of pro-inflammatory cytokines TNF-α, IL-6, and IL-1β mRNA levels. RAW 264.7 cells were treated for 6 h with the vehicle (DMSO; control) or 1 µg/mL LPS alone (control_LPS_) or with different sage extracts, at their respective IC_50_. Values are the mean ± SD of three independent experiments, each one performed in triplicate. *P*-values were calculated against control_LPS_; **** *p* < 0.0001.

The NF-κB pathway plays a key role in the maintenance of the inflammatory process as it promotes the transcription of various pro-inflammatory cytokines, an essential event for inflammatory stimulus propagation [41]. Based on the previous activity of extracts S1–S8 to inhibit the NF-κB pathway, we performed the quantitative polymerase chain reaction (PCR) analysis in order to evaluate the expression of tumor necrosis factor alpha (TNF-α), interleukin-6 (IL-6), and interleukin-1 beta (IL-1β), three important pro-inflammatory cytokines, at the transcript level. To this end, RAW 264.7 cells were stimulated with LPS and simultaneously exposed, for 6 h, to *S. officinalis* extracts S1–S8, at the respective IC_50_ values. The results presented in Figure 6 confirmed that the extracts exhibited a remarkable anti-inflammatory potential, underlined by the reduction in the expression levels of the inflammatory cytokines. In particular, extracts S2, S3, and S4 were the most active as they elicited a clear decrease in the expression levels of the three pro-inflammatory cytokines evaluated.

### 3.6. S. officinalis Extracts Interfere with Inflammation Associated with Neoplastic Progression

Inflammation is a physiological response triggered by the organism in order to overcome pathologic events including infection, or to repair tissue wounds. In this regard, it aims to defend the host organism [42]. However, dysregulated inflammatory mechanisms can lead to several diseases, including cancer [43].

In the past 20 years, inflammation has acquired an important role in cancer research as several neoplastic pathologies resulted from inflammatory processes transformed from physiological into pathological events, thus laying the foundation for the neoplastic process [43]. Several reports have demonstrated that some tumors are a direct consequence of severe inflammatory processes which, by subverting the tissue structure, potentially induce neoplasia. In recent years, there has been growing interest in the tumor microenvironment, as it is significantly involved in tumor progression. Literature data highlights the capacity of the tumor to release various factors such as cytokines and growth factors into the surrounding environment that are able to induce a systemic response, which in turn promotes tumor cell proliferation, survival, and migration [43]. Indeed, tumor cells liberate factors inducing new vessel formation in order to cope with the cancer tissue’s high demand for nutrients [44].

**Figure 7:**
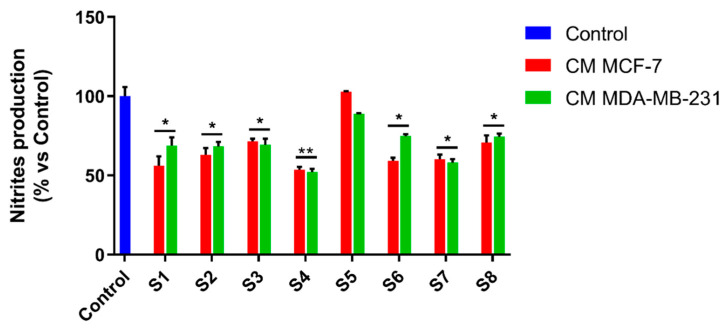
*Salvia officinalis* extracts decrease pro-inflammatory effects mediated by breast cancer cells conditioned media. Nitrites production was measured in RAW 264.7 cells after 24 h of treatment with conditioned media from MDA-MB-231 or MCF-7 cell lines (exposed before to IC_50_ values of S1–S8 extracts, for 24 h). Values are the mean ± SD of three independent experiments, each one performed in triplicate. *P*-values were calculated vs. control; * *p* < 0.05; ** *p* < 0.01.

The tumor microenvironment mostly contains fibroblasts and immune cells, including macrophages. Tumor-associated macrophages (TAMs) are M2-type macrophages surrounding malignant tumors whose presence is related to poor prognosis and survival [44]. It is known that breast cancer exhibits a large number of TAMs in the tumor microenvironment which interact and affect the surrounding cells that are involved in breast cancer progression and metastasis by several mechanisms. Therefore, therapies targeting these cells are under development and constitute a very attractive challenge for researchers [44]. Macrophages perform an action of considerable importance by phagocytizing and neutralizing pathogens foreign to our body. However, research by several teams has shown that the macrophages associated with a tumor are attracted to the tumor in response to the tumor’s release of various cytokines. In tumor tissue, macrophages do not have the ability to phagocytize tumor cells but release further pro-inflammatory factors, thus feeding inflammation and promoting tumor progression. In this context, we wondered whether *S. officinalis* extracts would be able to interfere with the inflammatory activity induced by breast cancer cell-conditioned media. To this end, we evaluated the ability of MCF-7 and MDA-MB-231 breast cancer cells to release factors able to activate macrophages in the culture medium. For this purpose, cancer cells were treated with S*alvia officinalis* extracts for 24 h. Then, cancer cell-conditioned media was collected, RAW 264.7 cells were exposed to it for 24 h, and NO production was assessed using the Griess assay, after exposure. The results displayed that the treatment with all the *S. officinalis* extracts (except S5) significantly decreased NO production (Figure 7), highlighting the reduced ability of cancer cell-conditioned media to elicit macrophage activation.

This result highlights, for the first time, the important role of *S. officinalis* extracts in controlling the inflammation processes related to neoplastic progression.

## 4. Conclusions

The study of plants and phytocomplexes that can be extracted from them is a central topic in the research of recent years. In this report, *Salvia officinalis* and, in particular, the extracts obtained by maceration or ultrasound in methanol of the sage, harvested in two different parts of Italy, were evaluated. The extraction methods used, although different from each other, have allowed us to obtain very similar chemical profiles with some differences. In detail, the extracts obtained by maceration proved to be more rich in TPC and TFC. Regardless of the extraction method used, it was possible to observe that the flowers contain more rosmarinic acid unlike the leaves which contain more luteolin-7-glucoside. The similar chemical profiles of Orsomarso and Civita sage extracts confer comparable activities in the four antioxidant assays. Indeed, the assays performed on the various extracts have shown a noticeable antioxidant effect (in ABTS and FRAP tests even better than the positive controls) regardless of the extraction method used, the part of the plant employed or the harvest area. These results confirm that *Salvia officinalis* is a rich source of antioxidant constituents. Moreover, our work aimed to evaluate if this high antioxidant potential results in beneficial effects on in vitro cellular models, focusing on the ability of sage extracts to modulate the oxidative stress induced by the inflammatory process. Indeed, since the close correlation between the inflammatory process and oxidative stress has been widely confirmed [12,19,43], the research is aimed at the continuous identification of compounds and phytocomplexes able to induce the subversion of this close connection. Our findings of in vitro antioxidant tests have been confirmed in LPS-stimulated murine macrophages RAW 264.7 cells that responded to the inflammatory stimulus with a substantial ROS production. The ROS increase was remarkably diminished when the cells were exposed simultaneously to the sage extracts, conferring on them the ability to reduce the oxidative stress, a representative feature of the inflammatory process. This result highlights, for the first time, the effect of *S. officinalis* extracts in modulating oxidative stress induced by an inflammatory process.

Inflammation is a physiological process able to respond to physical insults such as wounds or pathogens infections [43,45]. The control of this path becomes crucial as its exponential activation can make the process chronic with consequent associated routes induction such as excessive oxidative stress, initiation, and neoplastic progression [45]. For this purpose, compounds acting on this pathway are interesting as they can manifest different biological potentials.

The different sage extracts investigated in this study displayed, in general, a similar chemical profile and, consequently, comparable activity in the cell and in the inflammatory path modulation. However, Orsomarso extracts were slightly more active, in terms of IC_50_, in reducing the nitric oxide, one of the key markers of the inflammatory process.

Anti-inflammatory and antioxidant activities, albeit similar to each other, showed that the extract obtained after maceration in methanol of the sage leaves collected in Orsomarso is most active in reducing NO and ROS production. This activity could be due to the high concentration of luteolin-7-glucoside and to the good concentration in the extract of rosmarinic acid and flavonoids, both molecules considered by the literature endowed with anti-inflammatory and antioxidant properties [12,46,47,48]. Furthermore, our outcomes proved that the promising anti-inflammatory activity, is related to a decrease of NF-κB translocation, one of the main players in the inflammatory process and to the reduction of the main pro-inflammatory cytokines. Finally, given the possible involvement of the inflammatory path in the neoplastic process, both as a consequence or as an initiating cause of the same, we demonstrated the ability of *Salvia officinalis* extracts to modulate the pro-inflammatory effects of the typical feature of tumor cells. This evidence established, for the first time, that *Salvia officinalis* extracts are endowed with interesting potentiality in reducing inflammatory processes related to neoplastic disease.

This work on the whole, confirms the knowledge on the antioxidant potential *of Salvia officinalis* and broadens it at the same time, by laying the foundation for the use of sage extracts as interesting inhibitors of the inflammatory path associated with the neoplastic process, very often a cause of both its progression and greater aggressiveness.

## Figures and Tables

**Table 1:** The qPCR primers sequences.

Primer Name	Sequence (5′-3′)
TNFα Fw	CAGGCGGTGCCTATGTCTC
TNFα Rv	CGATCACCCCGAAGTTCAGTAG
IL-1β Fw	GAAATGCCACCTTTTGACAGTG
IL-1β Rv	TGGATGCTCTCATCAGGACAG
IL-6 Fw	CTGCAAGAGACTTCCATCCAG
IL-6 Rv	AGTGGTATAGACAGGTCTGTTGG
GAPDH Fw	ACCACAGTCCATGCCATCAC
GAPDH Rv	TCCACCACCCTGTTGCTGTA

**Table 2:** Extraction procedures and yield (%) of *S. officinalis* extracts (S1-S8).

*S. officinalis*		Part	Extraction Procedure	Yield (%) ^a^
Orsomarso	S1	Leaves	Maceration	7.8
	S2	Leaves	Ultrasound-assisted extraction	6.2
	S3	Flowers	Maceration	8.3
	S4	Flowers	Ultrasound-assisted extraction	3.8
Civita	S5	Leaves	Maceration	9.3
	S6	Leaves	Ultrasound-assisted extraction	8.4
	S7	Flowers	Maceration	8.9
	S8	Flowers	Ultrasound-assisted extraction	5.2

^a^ Expressed as [(g dried extract/g plant materials) × 100.

**Table 3:** Retention time, wavelengths of maximum absorption in the ultraviolet (UV)-visible (VIS) region, mass spectra data, and compounds identification in *S. officinalis*.

Peak	Rt (min)	UV λ (nm)	Molecular ion [M − H]^−^ (*m*/*z*)	Molecular Ion [M + H]^+^ (*m*/*z*)	Identification	Ref
1	11.5	220/240/295/325	179		Caffeic acid	[2]
2	18.4	205/253/270/350	447	449	Luteolin-7-glucoside^2^	[2]
3	19.6	208/253/270/345	461		Luteolin-*O*-glucuronide	[2]
4	20.8	220/251/290/330	359	361	Rosmarinic acid (cis,trans) ^2^	[1]
5	21.5	250/290/330	445		Apigenin-*O*-glucuronide	[2]
6	22.6	290/323	555	557	Salvianolic acid K	[3,4]
7	28.2	263/274/360	315		Nepetin	[2]
8	31.0	286/340	345	347	Rosmanol isomer ^1^	[1,2]
9	31.2	253/240/350	285		Luteolin	[2]
10	33.3	286/340	345	347	Rosmanol isomer ^1^	[1,2]
11	34.3	220/270/340	299		Hispidulin	[2]
12	34.8	286/340	345	347	Rosmanol isomer ^1^	[1,2]
13	36.3	279/340	313		Cirsimaritin	[2]
14	37.5	250/300/400	269		Apigenin	[2]
15	42.6	265/331	383		Genkwanin	[2]
16	42.8	205/290	359	361	7-*O*-methylrosmanol	[6]
17	45.7	205/250/305	342		Rosmadial	[2]
18	46.7	205/286	329	331	Carnosol ^2^	[1]
19	48.5	204/260/280/330	315	317	Pedalitin	[5]
20	49.9	205/230/285	345		12-*O*-methylcarnosic acid	[2]
21	50.4	204/235/287	331		Carnosic acid ^2^	[2]
22	54.5			457	Triterpenic acids (Ursolic acid) ^2^	[2]

^1^ Rosmanol, epirosmanol, epiisorosmanol, or/and isorosmanol. ^2^ Identification confirmed using commercial pure compounds.

**Table 4:** Luteolin-*7*-glucoside, rosmarinic acid, and triterpenic acids quantification in sage extracts by HPLC-DAD-Q-MS.

	Luteolin-7-Glucoside (Rt = 18.4 min)		Rosmarinic Acid (Rt = 20.8 min)		Triterpene Acids (Rt = 54.5 min)	
	(*)	(%) **	(*)	(%) **	(*)	(%) **
Orsomarso						
S1	0.0160 ± 0.0049	0.86	0.0848 ± 0.0128	4.56	0.2166 ± 0.0275	11.65
S2	0.0088 ± 0.0014	0.60	0.0514 ± 0.0093	3.50	0.3932 ± 0.0539	25.75
S3	0	0	0.0915 ± 0.0193	4.08	0.4851 ± 0.0511	21.66
S4	0.0024 ± 0.0005	0.12	0.1018 ± 0.0144	4.99	0.1574 ± 0.0017	7.72
Civita						
S5	0.0119 ± 0.0001	0.60	0.0654 ± 0.0077	3.30	0.1249 ± 0.0043	6.31
S6	0.0054 ± 0.0007	0.37	0.0559 ± 0.0005	3.83	0.1655 ± 0.0109	11.34
S7	0.0077 ± 0.0011	0.37	0.1127 ± 0.0106	5.44	0.0756 ± 0.0054	3.65
S8	0.0160 ± 0.0048	0.71	0.1750 ± 0.0440	7.81	0.1425 ± 0.0232	6.36

(*) Results are expressed as means values (g/L) ± standard deviation (*n* = 6); (**) % compound quantity injected versus extract quantity injected (*g*/*g*).

**Table 5:** Total phenols content (TPC), total flavonoids content (TFC), and antioxidant effects of *S. officinalis* S1–S8 extracts.

Sample	TPC ^1^	TFC ^2^	DPPH Test ^3^	ABTS Test ^3^	β-Carotene Bleaching Test ^3^		FRAP Test ^4^
					30 min	60 min	
S1	40.3 ± 1.5 ^b^	25.2 ± 1.4 ^b^	12.4 ± 1.2 ****	0.9 ± 0.08	7.1 ± 0.7 ****	11.6 ± 1.2 ****	98.4 ± 6.3
S2	18.8 ± 1.0 ^f^	12.6 ± 0.6 ^f^	10.4 ± 1.0 ****	0.9 ± 0.04	3.5 ± 0.3	3.2 ± 0.3	65.2 ± 4.2
S3	41.9 ± 1.6 ^a^	15.4 ± 0.9 ^d^	9.8 ± 0.9 ****	0.9 ± 0.09	2.9 ± 0.2	2.5 ± 0.2	95.5 ± 6.1
S4	25.7 ± 0.8 ^e^	14.5 ± 0.8 ^e^	10.8 ± 1.3 ****	1.1 ± 0.1	3.8 ± 0.4	4.3 ± 0.4 **	72.1 ± 4.4
S5	39.8 ± 1.5 ^c^	17.7 ± 0.9 ^c^	10.3 ± 1.0 ****	1.9 ± 0.2	5.1 ± 0.5 **	4.8 ± 0.4 **	69.1 ± 4.4
S6	35.3 ± 1.1 ^d^	14.9 ± 1.1 ^e^	11.8 ± 1.1 ****	1.1 ± 0.1	2.0 ± 0.2	2.5 ± 0.2	84.5 ± 5.1
S7	41.6 ± 1.2 ^a^	26.6 ± 0.7 ^a^	9.8 ± 0.9 ****	1.2 ± 0.1	4.0 ± 0.4 *	4.9 ± 0.5 **	87.1 ± 6.3
S8	18.7 ± 0.1 ^f^	12.4 ± 0.4 ^f^	11.8 ± 1.1 ****	0.9 ± 0.09	7.0 ± 0.7 ****	7.1 ± 0.7 ****	94.4 ± 6.3
Positive control							
Ascorbic acid			5.0 ± 0.8	1.7 ± 0.06			
Propyl gallate					1.0 ± 0.03	0.09 ± 0.004	
BHT							63.2 ± 4.3

^1^ Total phenols content (TPC): Mg chlorogenic acid (CA) equivalents/g plant materials; ^2^ Total flavonoids content (TFC): Mg quercetin (QE) equivalents/g plant materials; ^3^ IC_50_ (µg/mL) ^4^ μM Fe (II)/g (at a concentration of 1 mg/mL. Data are expressed as the mean ± standard deviation (*n* = 3). Means in the same column with different small letters differ significantly (*p* < 0.05). Differences within and between groups were evaluated by one-way ANOVA followed by a multi-comparison Dunnett’s test (α = 0.05): **** *p* < 0.0001, ** *p* < 0.01, * *p* < 0.1, compared with the positive controls in the biological assay, whereas multi-comparison Tukey’s test was applied for an evaluation of the phytochemical content (*p* < 0.05).

**Table 6:** Inhibitory activity on the nitrites production.

Extract	IC_50_ ± SD (µg/mL)
S1	21.74 ± 3.019
S2	31.92 ± 3.695
S3	44.43 ± 4.698
S4	25.02 ± 3.901
S5	49.83 ± 5.463
S6	22.95 ± 3.846
S7	28.87 ± 3.638
S8	58.83 ± 6.890

Data are presented as IC50 values (µg/mL) ± standard deviation (SD) obtained by nonlinear regression analysis of three independent experiments.
